# Clofarabine exerts antileukemic activity against cytarabine‐resistant B‐cell precursor acute lymphoblastic leukemia with low deoxycytidine kinase expression

**DOI:** 10.1002/cam4.1323

**Published:** 2018-02-23

**Authors:** Meixian Huang, Takeshi Inukai, Kunio Miyake, Yoichi Tanaka, Keiko Kagami, Masako Abe, Hiroaki Goto, Masayoshi Minegishi, Shotaro Iwamoto, Eiji Sugihara, Atsushi Watanabe, Shinpei Somazu, Tamao Shinohara, Hiroko Oshiro, Koshi Akahane, Kumiko Goi, Kanji Sugita

**Affiliations:** ^1^ Department of Pediatrics School of Medicine University of Yamanashi Yamanashi Japan; ^2^ Department of Health Sciences School of Medicine University of Yamanashi Yamanashi Japan; ^3^ Department of Clinical Pharmacy School of Pharmacy Kitasato University Tokyo Japan; ^4^ Hematology/Oncology and Regenerative Medicine Kanagawa Children's Medical Center Yokohama Japan; ^5^ Japanese Red Cross Fukushima Blood Center Fukushima Japan; ^6^ Department of Pediatrics Mie University Graduate School of Medicine Tsu Japan; ^7^ Division of Gene Regulation Institute for Advanced Medical Research School of Medicine Keio University Tokyo Japan

**Keywords:** Acute lymphoblastic leukemia, clofarabine, cytosine arabinoside, deoxycytidine kinase, drug resistance

## Abstract

Cytosine arabinoside (Ara‐C) is one of the key drugs for the treatment of acute myeloid leukemia. It is also used for consolidation therapy of acute lymphoblastic leukemia (ALL). Ara‐C is a deoxyadenosine analog and is phosphorylated to form cytosine arabinoside triphosphate (Ara‐CTP) as an active form. In the first step of the metabolic pathway, Ara‐C is phosphorylated to Ara‐CMP by deoxycytidine kinase (DCK). However, the current cumulative evidence in the association of the Ara‐C sensitivity in ALL appears inconclusive. We analyzed various cell lines for the possible involvement of DCK in the sensitivities of B‐cell precursor ALL (BCP‐ALL) to Ara‐C. Higher DCK expression was associated with higher Ara‐C sensitivity. DCK knockout by genome editing with a CRISPR‐Cas9 system in an Ara‐C‐sensitive‐ALL cell line induced marked resistance to Ara‐C, but not to vincristine and daunorubicin, indicating the involvement of DCK expression in the Ara‐C sensitivity of BCP‐ALL. *DCK* gene silencing due to the hypermethylation of a CpG island and reduced DCK activity due to a nonsynonymous variant allele were not associated with Ara‐C sensitivity. Clofarabine is a second‐generation deoxyadenosine analog rationally synthesized to improve stability and reduce toxicity. The IC50 of clofarabine in 79 BCP‐ALL cell lines was approximately 20 times lower than that of Ara‐C. In contrast to Ara‐C, although the knockout of DCK induced marked resistance to clofarabine, sensitivity to clofarabine was only marginally associated with *DCK* gene expression level, suggesting a possible efficacy of clofarabine for BCP‐ALL that shows relative Ara‐C resistance due to low DCK expression.

## Introduction

Cytosine arabinoside (Ara‐C; also known as cytarabine) is one of the key drugs for the treatment of acute myeloid leukemia (AML). It is also used for consolidation therapy of acute lymphoblastic leukemia (ALL). Ara‐C is a deoxycytidine analog that is phosphorylated into cytosine arabinoside triphosphate (Ara‐CTP) as an active form, which competes with deoxycytidine triphosphate (dCTP) for incorporation into DNA 1. In the first step of this metabolic pathway, Ara‐C is phosphorylated into Ara‐CMP by deoxycytidine kinase (DCK). Indeed, reduced DCK activity was demonstrated in the Ara‐C‐resistant erythroleukemic cell line, K562 2, and transfection of the *DCK* gene into Ara‐C‐resistant rat leukemic cell line restored in vitro Ara‐C sensitivity 3. In AML patients treated with Ara‐C, low *DCK* mRNA expression level was associated with a poor therapeutic outcome 4. The significance of DCK for Ara‐C sensitivity in ALL is rather controversial. Stammler et al. 5 reported that ALL patients with lower *DCK* gene expression relapsed more frequently than those with higher *DCK* gene expression. A recent single‐nucleotide polymorphism array analysis of the Ara‐C‐resistant xenograft model of ALL revealed that an Ara‐C‐resistant ALL subline, which spontaneously expanded during Ara‐C treatment, acquired a homozygous deletion of the *DCK* gene 6. These observations suggested that inactivation or low gene expression of DCK may be involved in Ara‐C resistance in ALL. In contrast, Stam et al. 7 reported that higher *DCK* gene expression tended to correlate with in vitro Ara‐C resistance in infant ALL.

Clofarabine (2‐chloro‐9‐[2‐deoxy‐2‐fluoro‐b‐D‐arabinofuranosyl] adenine) is a second‐generation deoxyadenosine analog rationally synthesized to improve stability and reduce the potential for dose‐limiting toxicity 8, 9. Following Food and Drug Administration approval for the use of clofarabine as a monotherapeutic agent for childhood refractory or relapsed ALL based on phase 1 and phase 2 studies 10, 11, combination therapy of clofarabine with other antileukemic agents revealed an encouraging outcome 12. Escherich et al. 13 reported that postinduction therapy consisting of clofarabine 5 × 40 mg/m^2^ and pegylated asparaginase (PEG‐ASP) 1 × 2500 iu/m^2^ was significantly more effective than standard therapy consisting of high‐dose Ara‐C 4 × 3 g/m^2^ and PEG‐ASP 1 × 2500 iu/m^2^ for newly diagnosed ALL patients. A significantly lower minimal residual disease level was found at the end of induction therapy with clofarabine, suggesting the antileukemic activity of clofarabine is clinically higher than that of Ara‐C. Clofarabine is phosphorylated to its monophosphate derivatives primarily by DCK 9. However, the potential relationship between the expression of DCK and the response to clofarabine in ALL is unknown 12.

In the present study, we tried to clarify the possible involvement of DCK in sensitivities to Ara‐C and clofarabine using a wide variety of B‐cell precursor ALL (BCP‐ALL) cell lines. Higher DCK expression was associated with higher Ara‐C sensitivity, and the knockout of DCK expression by a genome editing procedure using a CRISPR‐Cas9 system 14, 15 in an Ara‐C‐sensitive‐ALL cell line induced resistance to Ara‐C. In contrast, although the knockout of DCK also induced resistance to clofarabine, the sensitivity to clofarabine was only marginally associated with *DCK* gene expression. Our observations suggest efficacy of clofarabine for BCP‐ALL that shows relative resistance to Ara‐C due to low DCK expression.

## Materials and Methods

### Cell lines

Seventy‐nine BCP‐ALL cell lines were analyzed, which included 14 Philadelphia chromosome‐positive (Ph+) cell lines (KOPN30bi, 55bi, 56, 57bi, 66bi, 72bi, 83bi, YAMN73, 91, KCB1, Nalm27, SU‐Ph2, TCCS, SK9), 11 *MLL*‐rearranged (*MLL*+) cell lines (KOPN1, KOPB26, KOCL33, 44, 45, 50, 51, 58, 69, YACL95, RS4;11), 16 t(1;19)‐ALL cell lines (KOPNK, 34, 36, 54, 60, 63, YAMN90, 92, YCUB6, YCUB8, Kasumi2, THP4, SCMCL1, 697, RCH, PreALP), 4 t(17;19)‐ALL cell lines (UOC‐B1, HALO1, YCUB2, Endo‐kun), 3 t(12;21)‐ALL cell lines (KOPN41, 79, Reh), and 31 other cell lines (KOS20, KOPN32, 39, 35, 41, 49, 61, 62, 68, 70, 71, 75, 79, 84, 85, KCB4, YAMN74, THP5, 7, 8, YCUB4, 5, 7, MBKG, MBMY, MBOK, L‐ASK, L‐KUM, SCMCL2, P30/OHK, Nalm6). Seven BCP‐ALL cell lines (RS4;11, 697, RCH, PreALP, UOC‐B1, Reh, and Nalm6) were established from non‐Japanese patients, whereas seventy‐two BCP‐ALL cell lines were established from Japanese patients 16, 17, 18, 19, All cell lines were maintained in RPMI1640 medium supplemented with 10% fetal calf serum in a humidified atmosphere of 5% CO_2_ at 37°C.

### alamarBlue cell viability assay and detection of apoptosis

To determine the IC50s of Ara‐C and clofarabine, an alamarBlue cell viability assay (Bio‐Rad Laboratories, Hercules, CA) was performed; 1 − 4 × 10^5^ cells were plated into a 96‐well flat‐bottom plate and cultured in triplicate in the absence or presence of seven concentrations of each drug for 68 h. After a 6‐h additional incubation with alamarBlue, the absorbance at 570 nm was monitored by a microplate spectrophotometer using 600 nm as a reference wavelength. Cell survival was calculated by expressing the ratio of the optical density of treated wells to that of untreated wells as a percentage. The concentration of agent required to reduce the viability of treated cells to 50% of untreated cells was calculated, and the median of three independent assays was determined as IC50. For detection of apoptosis, cells were cultured in the absence or presence of Ara‐C or clofarabine for 48 h, stained with FITC‐conjugated Annexin V and 7‐aminoactinomycin D (7AAD) (MBL, Nagoya, Japan), and analyzed by flow cytometry (FACSCalibur, BD Biosciences, San Jose, CA).

### Real‐time RT‐PCR and sequencing of DCK

Total RNA was extracted using TRIzol reagent (Invitrogen, Carlsbad, CA), reverse transcription was performed using a random hexamer (Amersham Bioscience, Buckinghamshire, UK) and Superscript II reverse transcriptase (Invitrogen), and the sample was then incubated with RNase (Invitrogen). Real‐time reverse transcription (RT)–polymerase chain reaction (PCR) analyses of *DCK*,* ABCG2 (BCRP1), ENT1, ENT2, NT5C2, and DGUOK* were performed using Taqman probe kit (Hs01040726_m1, Hs01849026_s1, Hs01085706_m1, Hs01546959_g1, Hs01056741_m1, and Hs00361549_m1, respectively, Applied Biosystems, Foster City, CA). As an internal control, *ACTB* was quantified using Taqman RT‐PCR kit (Hs01060665_g1). For sequencing of the coding region of the *DCK* gene, 859‐bp region of exons 1–7, which contained 783 bp of entire open reading frame, was amplified with a forward primer (5′‐CCTCTTTGCCGGACGAGC‐3′) and a reverse primer (5′‐GGAACCATTTGGCTGCCTGT‐3′) and analyzed for direct sequencing with a reverse primer.

### Establishment of DCK knockout KOPN41 cells

To knockout DCK expression in KOPN41, an Ara‐C‐sensitive cell line established from t(12;21)‐ALL patient 19, we used a CRISPR‐Cas9 system 14, 15. We screened downstream sequence of initial ATG in exon 1 of the *DCK* gene using the CRISPR design tool (CRISPR DESIGN, http://crispr.mit.edu). We selected 5′‐atcaagaaaatctccatcgaagg‐3′, which showed the highest off‐target hit score, and the synthesized oligomers were cloned into CRISPR‐Cas9 vector (CRISPR CD4 Nuclease Vector, Thermo Fisher Scientific, Waltham, MA). After electroporation of the *DCK*‐targeting CRSIPR‐Cas9 vector using Neon electroporation transfection system (Thermo Fisher Scientific), the transfected cells were divided into 24‐well plates and cultured in the presence of 100 ng/mL of Ara‐C for 4 weeks. Untransfected cells were also divided into 24‐well plates and cultured in the same way. After the selection, genomic DNA was extracted from the Ara‐C‐resistant clones and amplified by PCR using a forward primer (5′‐GCCGCCACAAGACTAAGGA‐3′) and a reverse primer (5′‐GGAAAGCCCTCACCGAGTTG‐3′). The PCR products of clones #1 and #2 were sequenced using a TOPO TA‐cloning kit (Thermo Fisher Scientific). Protein expression of DCK was analyzed by immunoblotting, as previously reported 19, using anti‐DCK C‐terminal antibody (ABGENT, San Diego, CA) and anti‐Tubulin mouse antibody (SIGMA, Saint Louis, MO).

### Measurement of intracellular concentration of Ara‐CTP

Intracellular concentration of Ara‐CTP was measured by liquid chromatography–mass spectrometry (LC/MS). Triplicated samples of wild‐type and DCK knockout clone (#1) of KOPN41 cultured in the absence or presence of Ara‐C for 6 h were washed with PBS. Each frozen cell pellet (1.5 × 10^7^ cells) resuspended into 50 *μ*L of water with 1 *μ*L internal standard of ^13^C‐UTP (100 *μ*mol/L; Sigma‐Aldrich, St. Louis, MO) was vortexed for 30 sec and subsequently vortexed with 70% HPLC grade acetonitrile (Kanto Chemical, Tokyo, Japan) for 1 min. Five microliter of each supernatant of the sample mixture centrifuged at 13,200 × g for 10 min was applied into the LC‐MS/MS system composed of AQUITY H‐class ultra‐performance liquid chromatography and XevoTQD triple‐quadrupole tandem mass spectrometer (Waters, Milford, MA) and analyzed using MassLynx 4.1 software (Nihon Waters K.K. Tokyo, Japan). InertSustain AQC18 metal free PEEK column (150 mm × 2.1 mm, 3 *μ*m; GL Science, Tokyo, Japan) was used for separation. Column temperature and flow rate were set at 40°C and 0.3 mL/min, respectively, and autosampler temperature was set at 4°C. Gradient elution program was conducted for chromatographic separation with 5 mmol/L formic ammonium (Kanto Chemical, Tokyo, Japan) and acetonitrile as follows: 0 min (A 100%), 1.5 min (A 100%), 5.5 min (A10%, B90%), 9.5 min (A10%, B90%), and 10 min (A100%). The mass spectrometry was operated in the ESI positive mode and quantified by multiple reactions monitoring (MRM). Nitrogen gas at 350°C with 1000 L/h of flow rate and argon gas with 50 L/h of flow rate were used as desolvation and collision, respectively. Capillary voltage was set at 3.5 kV. The MRM transitions were used for the quantification as follows: 483.99 to >112.01 for Ara‐CTP with collision energy of 20 V and 493.92 to >101.95 for ^13^C‐Uridine triphosphate with collision energy of 24 V. Retention time for Ara‐CTP and ^13^C‐UTP were 0.95 and 0.91 min, respectively. Calibration curve of standard Ara‐CTP (Jena Bioscience, Jena, Germany) at the concentrations of 0.5–2000 nmol/mL was *y* = 0.0020 + 0.6612 (*r* ^2 ^= 0.995).

### Methylation analysis by bisulfite sequencing

Genomic DNA was subjected to sodium bisulfite modification with an EZ DNA Methylation‐Lightning kit (Zymo Research, Irvine, CA). Bisulfite‐modified DNA was amplified for a CpG island of the *DCK* gene by PCR with a forward primer (5′‐ GTTGTTTGGGGTAGAGGTTTTT‐3′) and a reverse primer (5′‐ CTAAACCAAATCCTAACCTACC‐3′) using one cycle of 95°C for 4 min, 40 cycles of 95°C for 30 sec, 55°C for 30 sec, 72°C for 1 min, and with a final cycle of 72°C for 7 min. Bisulfite‐modified DNA of two cell lines (KOPN66bi and Kasumi2) was also amplified for a CpG island of the *BIM* gene 20 as positive control with a forward primer (5′‐TTTTTAAATGTTTGATTTTGATTTT‐3′) and a reverse primer (5′‐ACCAAAAACCTACAAATTCC‐3′) 21. For a next‐generation sequencing, amplicon libraries were generated by an Ion Plus Fragment Library Kit (Thermo Fisher Scientific; MAN0006846) and Ion Xpress Barcode Adaptors Kit (Thermo Fisher Scientific). Following Agencourt AMPure XP purification (Beckman Coulter, Brea, CA), individual libraries were quantified by quantitative real‐time PCR then diluted and finally pooled in equimolar ratios. The libraries were processed with an Ion OneTouch^™^ 2 System using an Ion PGM^™^ Template OT2 400 Kit (Thermo Fisher Scientific) to produce 400 base‐read libraries. Sequencing was performed using an Ion PGM^™^ Hi‐Q Sequencing Kit (Thermo Fisher Scientific) and 850 flows on an Ion 318 Chip Kit v2 (Thermo Fisher Scientific) according to the manufacturer's protocol. After sequencing, single processing and base‐calling were performed using Torrent Suite 5.0.2 (Thermo Fisher Scientific). Methylation analysis was performed using Methylation Analysis_Amplicon plug‐in v1.3 (Thermo Fisher Scientific).

### Statistics

Mann–Whitney test was applied for comparison of drug sensitivities, chi‐square test was for comparison of deletion incidence, a paired *t*‐test was for comparison in the same subject, and Pearson's correlation analysis was for correlation between two factors using Excel software (Microsoft, Redmond, WA).

## Results

### Ara‐C sensitivity in 79 BCP‐ALL cell lines

**Figure 1:**
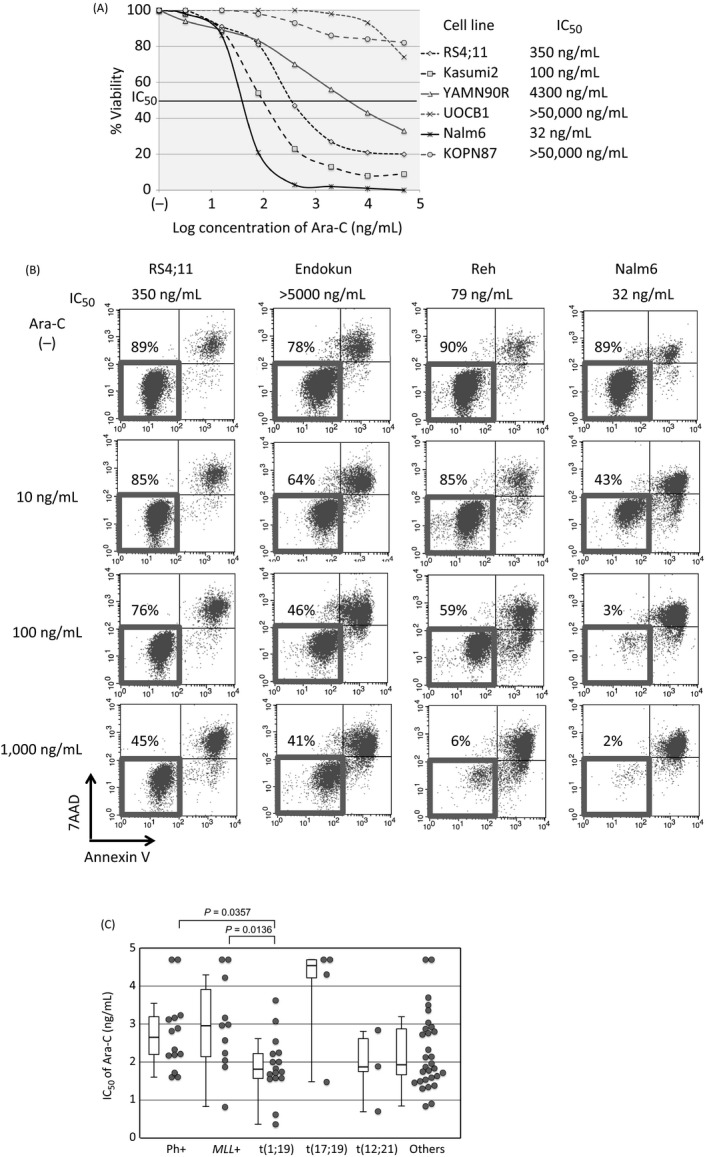
Ara‐C sensitivity in BCP‐ALL cell lines. (A) Dose–response curves of Ara‐C. Representative results of alamarBlue assays are indicated. The vertical axis indicates the % viability, and the horizontal axis indicates the log concentration of Ara‐C (ng/mL). (B) Induction of apoptotic cell death by Ara‐C. Cell lines were cultured in the absence or presence of 10, 100, and 1000 ng/mL of Ara‐C for 48 h and analyzed with Annexin V‐binding (horizontal axis) and 7AAD‐staining (vertical axis) using flow cytometry. The percentages of living cells are indicated. (C) Comparison of Ara‐C sensitivity among five representative chromosomal translocations in 79 BCP‐ALL cell lines. Statistically significant differences determined by Mann–Whitney tests are indicated with a *P* value.

We, firstly, analyzed Ara‐C‐sensitivity of 79 BCP‐ALL cell lines using an alamarBlue assay. Based on a dose–response curve in triplicate experiments (Fig. 1A), the median of 50% inhibitory concentrations in three independent analyses was scored as IC50 for each cell line. The induction of apoptotic cell death was confirmed in representative cell lines by flow cytometric analyses with 7AAD‐staining and Annexin V‐binding (Fig. 1B). We evaluated an association between the types of translocation and Ara‐C sensitivity. As shown in Figure 1C, t(1;19)‐ALL cell lines (median IC50; 77 ng/mL) were significantly more sensitive than Ph+ALL cell lines (435 ng/mL, *P *=* *0.0357 in a Mann–Whitney test) and *MLL*+ALL cell lines (960 ng/mL, *P *=* *0.0136). Although statistically not significant probably due to limited numbers of cell lines, t(17;19)‐ALL cell lines (35,000 ng/mL) were relatively resistant, whereas t(12;21)‐ALL cell lines (79 ng/mL) were relatively sensitive.

### Association of *DCK* gene expression with Ara‐C sensitivity

**Figure 2:**
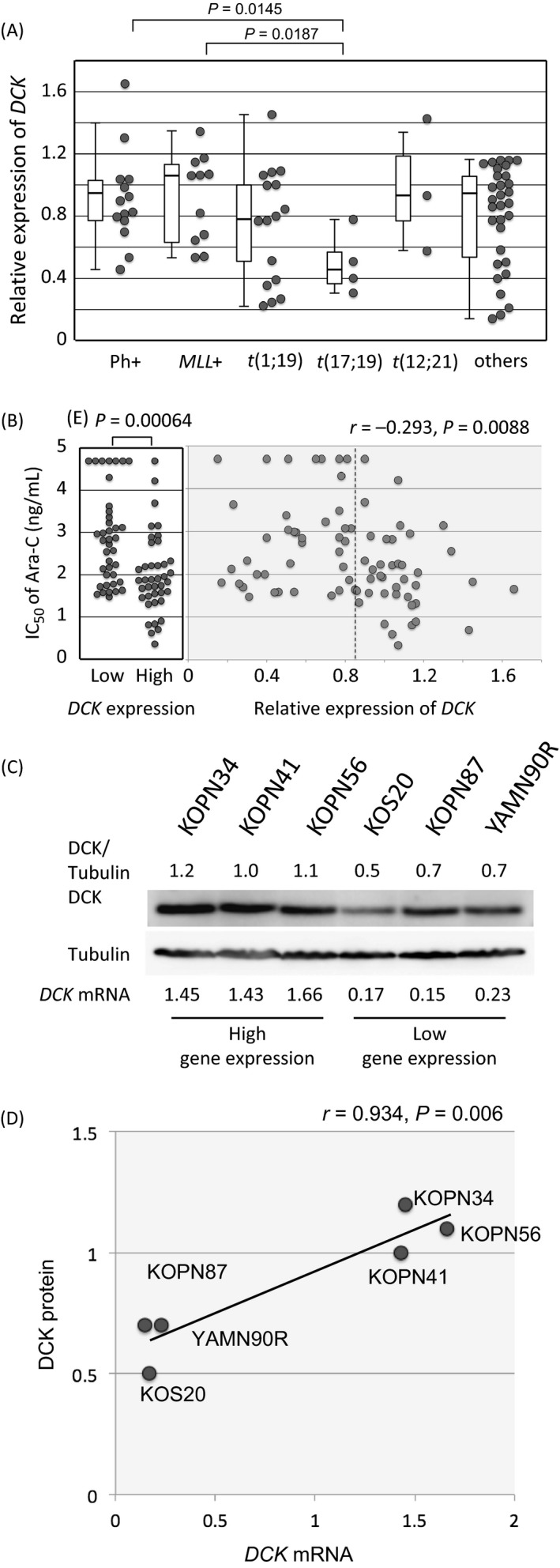
Deoxycytidine kinase expression and Ara‐C sensitivity in BCP‐ALL cell lines. (A) A comparison of *DCK* gene expression level among five representative chromosomal translocations in 79 BCP‐ALL cell lines as determined by RT‐PCR. Statistically significant differences in the Mann–Whitney test are indicated with a *P* value. (B) Association of Ara‐C sensitivity with *DCK* gene expression in 79 BCP‐ALL cell lines. In the left panel, IC50 values are compared between 40 and 39 cell lines with relatively higher and lower *DCK* gene expressions, respectively. Dotted line indicates median *DCK* gene expression level. In the right panel, the association between the IC50 of Ara‐C (vertical axis) and *DCK* gene expression (horizontal axis) is indicated. (C) The DCK protein expression level in BCP‐ALL cell lines. The DCK protein expression level was analyzed by immunoblotting in three cell lines with high *DCK* gene expression (KOPN34, KOPN41, and KOPN56) and in three cell lines with low *DCK* gene expression (KOS20, KOPN87, and YAMN90R) using tubulin expression as an internal control. The relative mRNA expression of the *DCK* gene is indicated in the bottom of the panel. The relative DCK protein expression is indicated at the top of the panel. (D) Correlation between the DCK protein expression level (vertical axis) and *DCK* gene expression (horizontal axis) in six BCP‐ALL cell lines.

To verify a possible association of *DCK* gene expression with Ara‐C sensitivity 2, 3, 4, 5, 6, we quantified the gene expression of *DCK* in 79 BCP‐ALL cell lines by real‐time RT‐PCR. Gene expression of *DCK* in t(17;19)‐ALL cell lines (median relative expression level; 0.46) was significantly lower than that in Ph+ALL cell lines (0.87, *P *=* *0.0145 in a Mann–Whitney test) and *MLL+*ALL cell lines (1.07, *P *=* *0.0187; Fig. 2A). We simply compared Ara‐C sensitivity between 40 cell lines with relatively higher *DCK* gene expression (equal to or higher than median level of 79 cell lines) and 39 cell lines with lower *DCK* gene expression (lower than median level; Fig. 2B). Of note, 40 cell lines with higher *DCK* gene expression were significantly more sensitive to Ara‐C (*P* = 0.00064; median IC50, 71 ng/mL) than 39 cell lines with lower *DCK* gene expression (700 ng/mL); however, the gene expression of *DCK* was not significantly correlated with the IC50 of Ara‐C (*r *=* *−0.293). We next investigated an association between the gene and protein expression of DCK in six representative BCP‐ALL cell lines, three cell lines each with high and low *DCK* gene expression (Fig. 2C). The protein expression level of DCK in the three cell lines showing high *DCK* gene expression was significantly higher than that in the three cell lines with low gene expression, and a significant correlation was observed between the gene and protein expression of DCK in six cell lines (*r *=* *0.934, *P *=* *0.0006; Fig. 2D).

### Knockout of DCK by CRISPR‐Cas9 system

**Figure 3:**
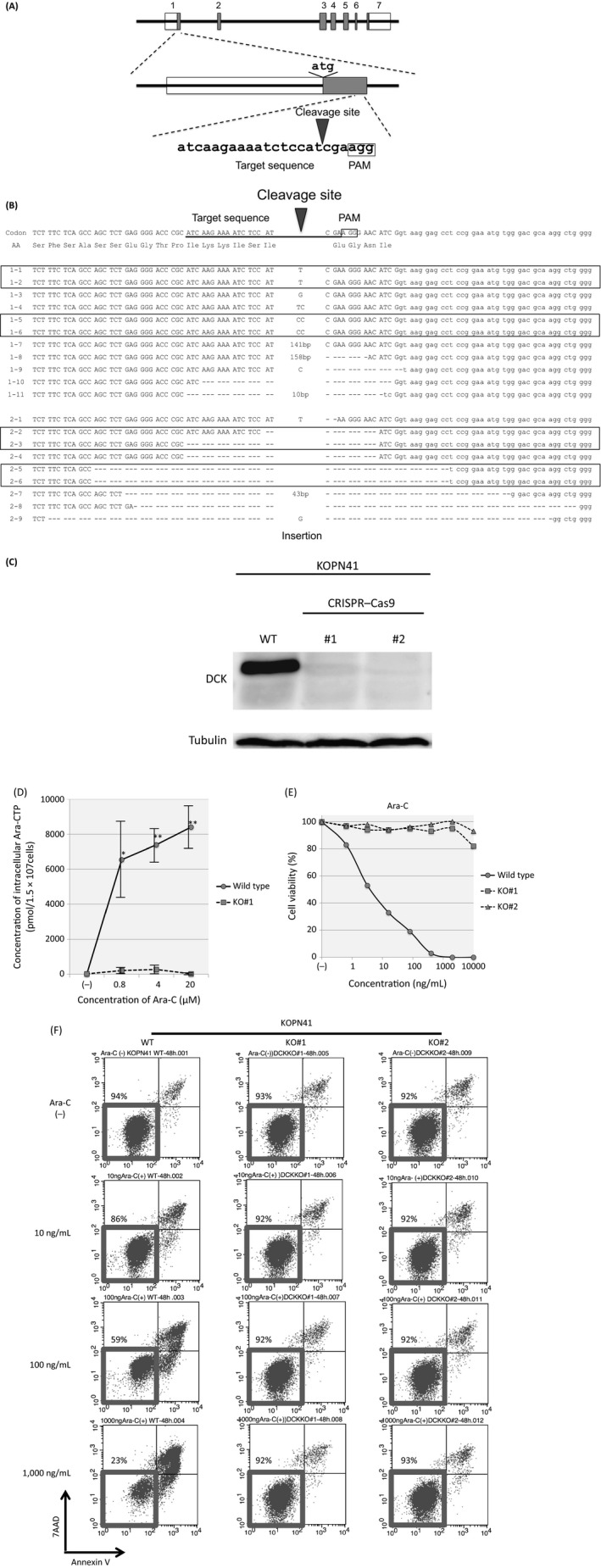
Knockout of DCK using a CRISPR‐Cas9 system in KOPN41 cells. (A) Schematic representation of the target sequence of CRISPR‐Cas9. (B) Sequence of the cleavage site in clones #1 and #2. Boxes indicate identical sequences. (C) DCK protein expression in knockout clones and wild‐type (WT) cells as determined by immunoblotting. (D) Intracellular concentrations of Ara‐CTP in wild‐type and DCK knockout (KO) clone of KOPN41. Cells were cultured in the absence or presence of 0.8 (195), 4 (973), and 20 *μ*mol/L (4864 ng/mL) of Ara‐C for 6 h, and intracellular Ara‐CTP was measured by liquid chromatography–mass spectrometry (LC/MS). The vertical axis indicates intracellular concentration of Ara‐CTP (pmol/1.5 × 10^7^ cells), and the horizontal axis indicates the concentration of Ara‐C (*μ*mol/L). Error bars indicate standard error of triplicate experiment. Asterisks indicate significance (*P < 0.05, **P < 0.01) in a paired *t*‐test between wild‐type cells and DCK KO cells at each concentration of Ara‐C. (E) Dose–response curve of Ara‐C in wild‐type and DCK knockout (KO) clones of KOPN41. Representative results of alamarBlue assays are indicated. The vertical axis indicates the % viability, and the horizontal axis indicates the log concentration of Ara‐C (ng/mL). (F) The induction of apoptotic cell death by Ara‐C. Cells were cultured in the absence or presence of 10, 100, and 1000 ng/mL of Ara‐C for 48 h and analyzed with Annexin V‐binding (horizontal axis) and 7‐aminoactinomycin D (7AAD) viability staining (vertical axis) using flow cytometry. The percentages of living cells are indicated.

The above observations indicated that the DCK expression level is associated with Ara‐C sensitivity in BCP‐ALL cell lines. To directly verify the involvement of DCK in the antileukemic activity of Ara‐C in BCP‐ALL, we tried to knockout DCK expression by a genome editing procedure using a CRSIPR‐Cas9 system 14, 15 in KOPN41, an Ara‐C‐sensitive cell line with high DCK expression. We targeted a downstream sequence of the initial ATG codon in exon 1 of the *DCK* gene (Fig. 3A), which showed the highest off‐target hit score by a CRISPR design tool (score 93). After electroporation of the *DCK*‐targeting CRSIPR‐Cas9 vector, transfected and untransfected control cells were separately divided into 24‐well plates and cultured in the presence of 100 ng/mL of Ara‐C. Four weeks later, all of the untransfected control cells were killed completely, while the Ara‐C‐resistant clones were selected in seven (29.2%) of 24 wells of the CRISPR‐Cas9‐transfected cells. We performed PCR of the genomic DNA extracted from the Ara‐C‐resistant clones using primers that were designed at both sides of the target sequence. Electrophoresis of the PCR products revealed extra products with abnormal sizes in all of the seven clones (Fig. S1). The PCR products of clones #1 and #2 were subcloned into a TA‐cloning vector and sequenced. The PCR products revealed nine and seven patterns of insertion or deletion 3 bp upstream of the PAM site in clones #1 and #2, respectively (Fig. 3B). A wild‐type sequence was not obtained in either clone, indicating that both alleles of the DCK gene were mutated by the CRISPR‐Cas9 system. As a result, DCK protein expression was almost undetectable in both clones by immunoblot analysis (Fig. 3C). We next measured intracellular concentration of Ara‐CTP in both wild‐type and DCK knockout clone (#1) of KOPN41, which were cultured in the absence or presence of 0.8–20 *μ*mol/L (195–4864 ng/mL) of Ara‐C for 6 h, using liquid chromatography–mass spectrometry (LC/MS). As shown in Figures 3D and S2, intracellular Ara‐CTP was accumulated in the wild‐type cells in a dose‐dependent manner, while it was almost undetectable in the DCK knockout clone. These observations demonstrated that DCK is indispensable for the activation Ara‐C at least in KOPN41 cells.

### Ara‐C‐specific resistance in a DCK knockout ALL cell line

We investigated Ara‐C sensitivity in two knockout clones (#1 and #2) of KOPN41 in comparison with parental KOPN41 cells using an alamarBlue assay (Fig. 3E) and flow cytometric analysis (Fig. 3F). Of note, both of the knockout clones were highly resistant to Ara‐C (Fig. 3E). The cell viabilities of knockout clones were approximately 90% after 48‐h culture in the presence of 1000 ng/mL of Ara‐C (Fig. 3F), whereas that of parental cells was approximately 20%. To verify the Ara‐C‐specific resistance of the DCK knockout clones, we next investigated sensitivities to vincristine (VCR) and daunorubicin (DNR). In contrast to Ara‐C sensitivity, the knockout clones were as sensitive to VCR and DNR as parental cells, as determined in the alamarBlue assay (Fig. S3A) and by flow cytometric analysis (Fig. S3B and C), indicating the acquisition of Ara‐C‐specific resistance in the DCK knockout KOPN41 cells.

### Methylation of *DCK* gene promoter

**Figure 4:**
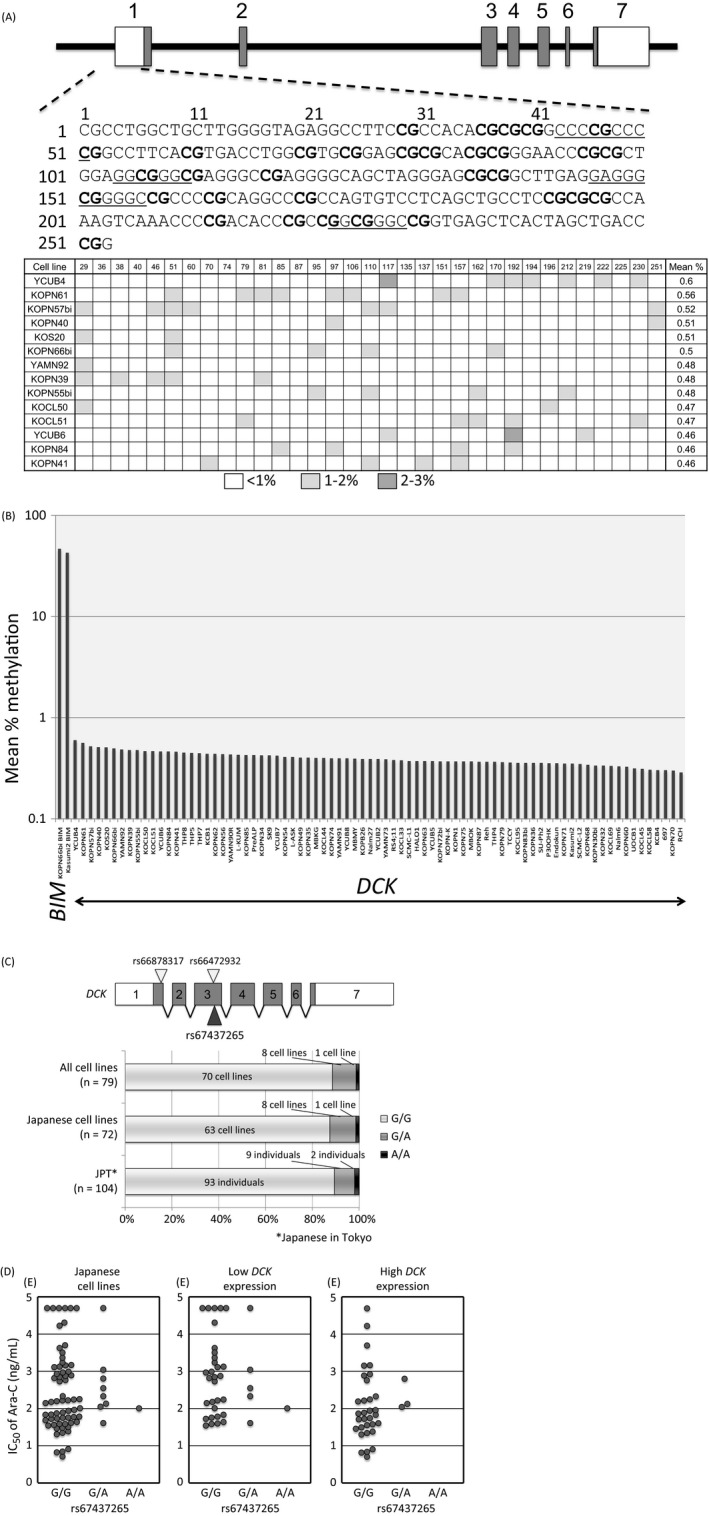
Methylation status of CpG island and genotypes of nonsynonymous single‐nucleotide polymorphisms in the *DCK* gene. (A) Schematic representation of a CpG island and DNA methylation of the *DCK* gene. Potential SP1 binding sites are underlined. A heat map of the methylation status in each of 33 CG dinucleotide sites located in the promoter is shown. The mean % methylation in 33 CG dinucleotide sites in each cell line is shown on the right side of the lower panel. (B) Mean % methylation in the promoter region of the *DCK* gene in 79 BCP‐ALL cell lines as determined by bisulfite sequencing. As a positive control, the mean % methylation of 27 CG dinucleotide sites in the CpG island of the *BIM* gene that was determined using identical bisulfite‐treated genomic DNA of two cell lines (KOPN66bi and Kasumi2) is indicated. (C) Schematic representation of three nonsynonymous single‐nucleotide polymorphisms (SNPs) in the *DCK* gene and frequencies of the rs67437265 genotype in 79 BCP‐ALL cell lines, and in 72 BCP‐ALL cell lines established from Japanese patients in comparison with those in 104 normal Japanese individuals reported in the 1000‐genome project database. (D) The association of the rs67437265 genotype with Ara‐C sensitivity in 72 BCP‐ALL cell lines established from Japanese patients (left), 37 BCP‐ALL cell lines established from Japanese patients with relatively low *DCK* gene expression (middle), and 35 BCP‐ALL cell lines established from Japanese patients with relatively high *DCK* gene expression (right).

A CpG island that contains four potential SP1 binding sites exists in the promoter region of the *DCK* gene 22. Thus, we hypothesized that DNA methylation of the CpG island may affect *DCK* gene expression and subsequently Ara‐C sensitivity. To verify this possibility, we sequenced the CpG island of the *DCK* gene after bisulfite treatment of genomic DNA. After PCR amplification of the region, which contains 33 CG dinucleotide sites, we determined the sequence using a next‐generation sequencer and quantified the % methylation of each CG dinucleotide site. As representatively shown in Figure 4A, % methylation was always <3% at each of 33 CG dinucleotide sites in all of the 79 BCP‐ALL cell lines. The mean % methylation of the 33 CG dinucleotide sites in the CpG island of the *DCK* gene was <1% in all cell lines (Fig. 4B). In contrast, when 27 CG dinucleotide sites in the CpG island of the *BIM* gene 20, 21 were analyzed as a positive control using the identical bisulfite‐treated genomic DNA of two cell lines (KOPN66bi and Kasumi2), the mean % methylation was higher than 40% in both cell lines (Fig. 4B). These observations indicated that hypermethylation of the CpG island is not involved in the silencing of the *DCK* gene in BCP‐ALL cell lines.

### Somatic mutation and polymorphisms in the coding exons of the *DCK* gene

Considering the association of *DCK* gene expression with Ara‐C sensitivity in BCP‐ALL cell lines and Ara‐C‐specific resistance in DCK knockout cells, we speculated that somatic mutations in the *DCK* gene may be involved in Ara‐C resistance in BCP‐ALL cell lines. Thus, we directly sequenced seven coding exons of the *DCK* gene in 79 BCP‐ALL cell lines, but a somatic mutation was not observed in these cell lines (data not shown). Next, we focused on three previously known nonsynonymous single‐nucleotide polymorphisms (SNPs) of the *DCK* gene, rs66878317 (I24V), rs66472932 (A119G), and rs67437265 (P122S) 23. Although no variant allele was observed at rs66878317 and rs66472932, a variant allele of rs67437265 was observed in nine cell lines (Fig. S4). Among 72 cell lines established from Japanese patients, eight (11.1%) cell lines and one cell line (1.4%) showed a heterozygous and homozygous variant allele, respectively. The frequency of the variant allele was 6.9% (10/144 alleles), which was almost identical to the frequency in the normal Japanese population (6.3%; 13/208 alleles) reported in the 1000‐genome project database (Fig. 4C). To verify the impact of the substitution of Ser122 to Pro on Ara‐C sensitivity, we analyzed the association of rs67437265 genotype with Ara‐C sensitivity in 72 BCP‐ALL cell lines established from Japanese patients (Fig. 4D). The median IC50 value of Ara‐C was 163 and 340 ng/mL in 63 wild‐type cell lines and nine cell lines with a variant allele, respectively. A statistically significant association was not observed between the two populations in 72 cell lines, as well as in 37 and 35 cell lines with relatively lower and higher *DCK* gene expression, respectively.

### Association of gene expression of regulatory factors with Ara‐C sensitivity

**Figure 5:**
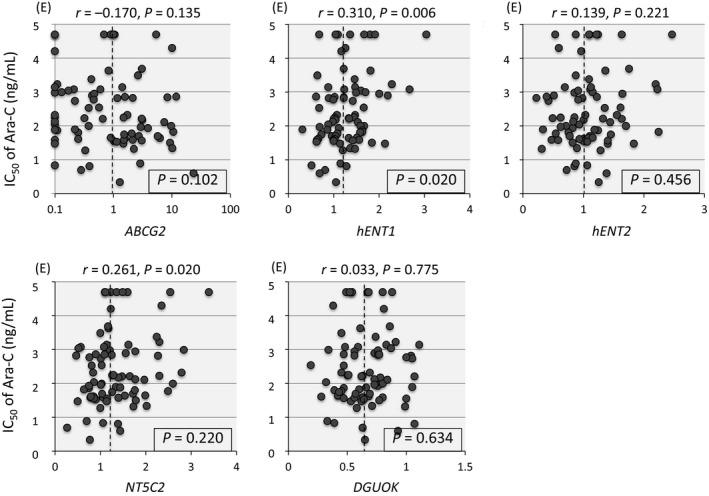
Association of Ara‐C sensitivity with gene expression of *ABCG2* (*BCRP1*), *hENT1*,* hENT2*,* NT5C2,* and *DGUOK* in 79 BCP‐ALL cell lines. In each panel, vertical axis indicates log IC50 value of Ara‐C, while horizontal axis indicates relative gene expression level. The correlation coefficient and *P* value are indicated at the top of each panel. Dotted line indicates median gene expression level. *P* value in a Mann–Whitney test of IC50 between 40 and 39 cell lines with relatively higher and lower gene expressions, respectively, is indicated in box.

Previous reports revealed possible associations of transporters such as ABCG2 (BCRP1) 24, hENT1 (SLC29A1) 1, and hENT2 (SLC29A2) 25, activating factor such as deoxyguanosine kinase (DGUOK) 26, and inactivating factors such as 5′ nucleotidase (NT5C2) 1 and cytidine deaminase (CDA) 1 with Ara‐C sensitivity of leukemia cells. Thus, we tried to analyze association between the gene expression levels of these six factors and the sensitivity to Ara‐C in 79 BCP‐ALL cell lines (Fig. 5). Among three transporters tested, gene expression levels of *ABCG2* (an efflux membrane transporter) and *hENT2* (an uptake transporter) were not associated with Ara‐C sensitivity. Unexpectedly, cell lines with relatively higher *hENT1* (an uptake transporter) gene expression were more resistant to Ara‐C than those with lower expression (*P *=* *0.020 in a Mann–Whitney test), although statistically significant correlation (*R* ^2 ^> 0.2) was not observed between the gene expression of *hENT1* and the IC50 of Ara‐C (*r *=* *0.310). Gene expression level of *DGUOK* was not associated with Ara‐C sensitivity. Among inactivating factors, an association of *CDA* gene was not evaluable due to low or undetectable expression. Gene expression level of *NT5C2* was not associated with Ara‐C sensitivity.

### Clofarabine sensitivity in 79 BCP‐ALL cell lines

**Figure 6:**
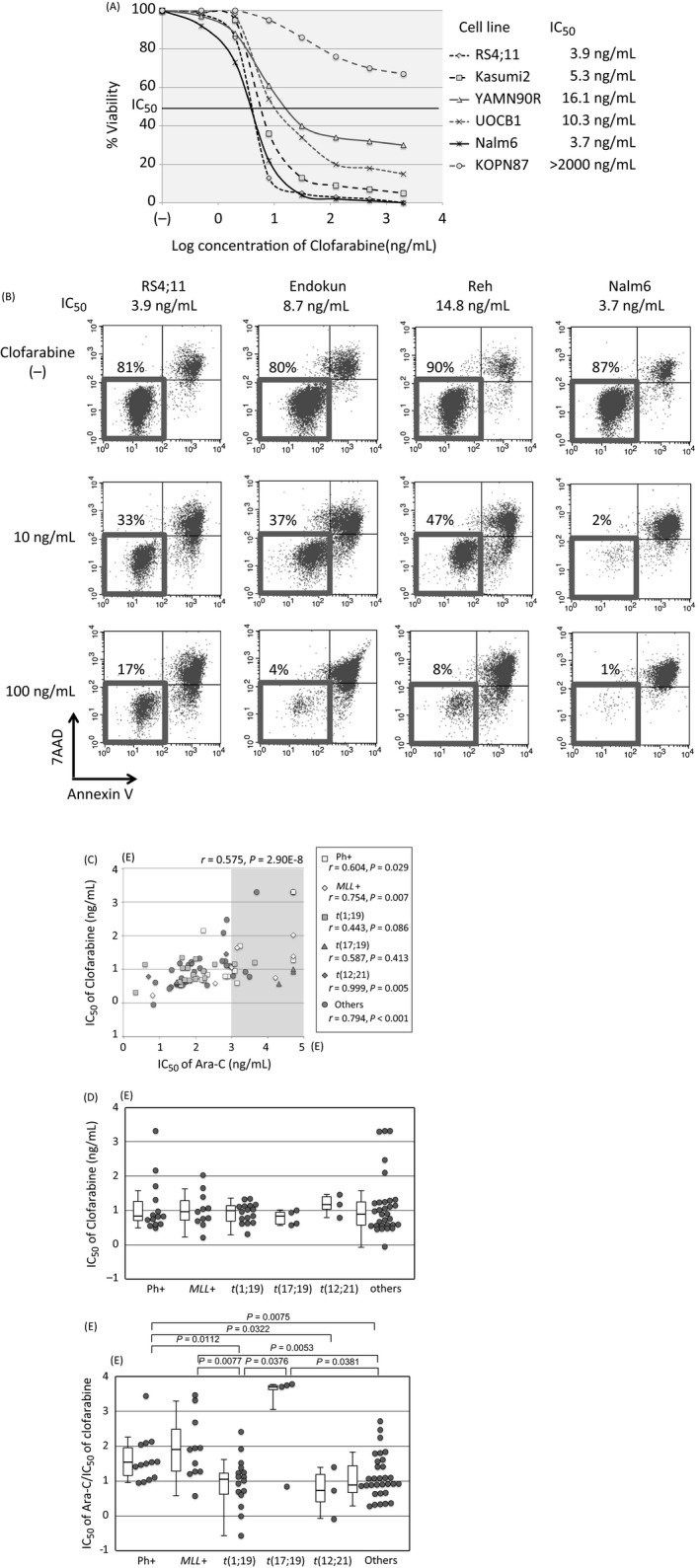
Sensitivity to clofarabine in BCP‐ALL cell lines. (A) Dose–response curves for clofarabine. Representative results of alamarBlue assays are indicated. The vertical axis indicates the % viability, and the horizontal axis indicates the log concentration of clofarabine (ng/mL). (B) The induction of apoptotic cell death by clofarabine. Cells were cultured in the absence or presence of 10 and 100 ng/mL of clofarabine for 48 h and analyzed by flow cytometry. The percentages of living cells are indicated. (C) Correlation between IC50 of Ara‐C (horizontal axis) and IC50 of clofarabine (vertical axis) is indicated. Correlation coefficients and *P* values are indicated on the top, and those for each of five representative chromosomal translocations are indicated in the box. (D) A comparison of clofarabine sensitivity in 79 BCP‐ALL cell lines among five representative chromosomal translocations. (E) A comparison of the ratio between the IC50 of Ara‐C and clofarabine among five representative chromosomal translocations. Statistically significant differences in Mann–Whitney tests are indicated with *P* values.

We next evaluated sensitivity to clofarabine using alamarBlue assays (Fig. 6A). The induction of apoptotic cell death in a dose‐dependent fashion was confirmed in representative cell lines by flow cytometric analyses (Fig. 6B). As shown in Figure 6C, a significant positive correlation was observed between the IC50 of clofarabine and the IC50 of Ara‐C (*r *=* *0.575, *P *=* *2.9 × 10^−8^). Correlation was significant in Ph+ALL, *MLL*+ALL, t(12;21)‐ALL, and other ALL cell lines, while it was insignificant in t(1;19)‐ALL and t(17;19)‐ALL cell lines. The median IC50s of clofarabine and Ara‐C were 8.5 and 163 ng/mL, respectively. The IC50 of clofarabine was approximately 20 times lower than that of Ara‐C. Among 20 Ara‐C‐resistant cell lines, whose IC50 of Ara‐C was 1000 ng/mL or higher, 12 cell lines (60%) were sensitive to clofarabine (IC50 of clofarabine <20 ng/mL). Although clofarabine sensitivity was correlated significantly to Ara‐C sensitivity, no significant association was observed between the types of translocation and the IC50s of clofarabine (Fig. 6D). We, subsequently, evaluated the ratio of the IC50 of Ara‐C divided by that of clofarabine (Fig. 6E). Of note, the ratio was significantly higher in cell lines with poor prognostic translocations such as Ph+ALL (median ratio; 32.1), *MLL*+ALL (79.3), and t(17;19)‐ALL cell lines (5000) than that in cell lines having favorable translocations such as t(1;19)‐ALL cell lines (11.2) and t(12;21)‐ALL cell lines (5.3), as well as other BCP‐ALL cell lines (8.2).

### Association of *DCK* gene expression with clofarabine sensitivity

**Figure 7:**
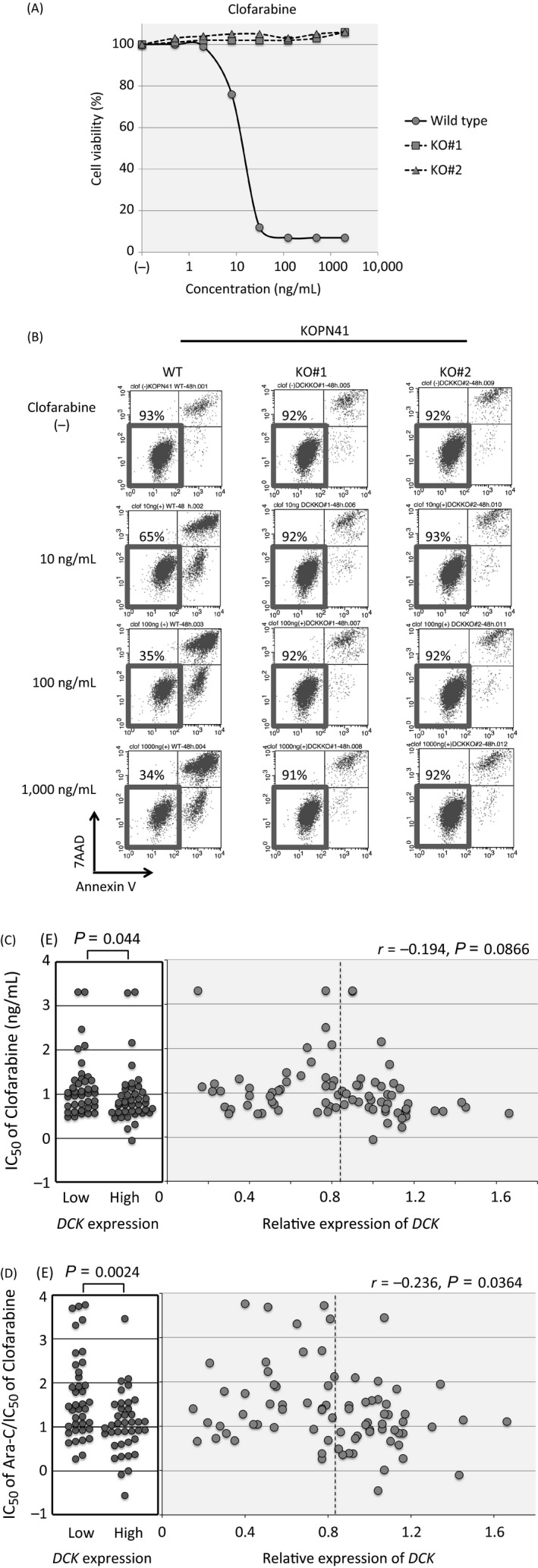
Association of *CDK* gene expression with clofarabine sensitivity of BCP‐ALL cell lines. (A) Dose–response curves for clofarabine in wild‐type and DCK knockout (KO) clones of KOPN41. Representative results of alamarBlue assays are indicated. The vertical axis indicates the % viability, and the horizontal axis indicates the log concentration of clofarabine (ng/mL). (B) The induction of apoptotic cell death by clofarabine. Cells were cultured in the absence or presence of 10, 100, and 1000 ng/mL of clofarabine for 48 h and analyzed with Annexin V‐binding (horizontal axis) and a 7AAD stain (vertical axis) using flow cytometry. The percentages of living cells are indicated. (C) The association of clofarabine sensitivity with DCK expression in 79 BCP‐ALL cell lines. In the left panel, IC50 values are compared between 40 cell lines with relatively higher *DCK* gene expression and 39 cell lines with lower *DCK* gene expression. Dotted line indicates median *DCK* gene expression level. In the right panel, an association between the IC50 of clofarabine (vertical axis) and *DCK* gene expression level (horizontal axis) is indicated. (D) Association of the ratio between the IC50 of Ara‐C and the IC50 of clofarabine with DCK expression in 79 BCP‐ALL cell lines. In the left panel, the ratios between the IC50 of Ara‐C and clofarabine are compared between 40 and 39 cell lines with relatively higher and lower *DCK* gene expressions, respectively. Dotted line indicates median *DCK* gene expression level. In the right panel, the association between ratios of the IC50 of Ara‐C and clofarabine (vertical axis) and *DCK* gene expression (horizontal axis) is indicated.

We investigated the sensitivity to clofarabine in DCK knockout clones of KOPN41 in comparison with parental KOPN41 cells. Both of the knockout clones were highly resistant to clofarabine in the alamarBlue assay (Fig. 7A). The cell viabilities of knockout clones were approximately 90% after a 48‐h culture in the presence of 100 and 1000 ng/mL of clofarabine, whereas that of the parental cells was approximately 30% (Fig. 7B). These observations indicated that DCK expression is indispensable for the antileukemic activity of clofarabine. However, the association of clofarabine sensitivity with *DCK* gene expression in 79 BCP‐ALL cell lines was marginal (Fig. 7C); the IC50 values of clofarabine in 40 cell lines with relatively higher *DCK* gene expression (median IC50; 6.3 ng/mL) were slightly lower (*P *=* *0.044) than those in 39 cell lines with lower *DCK* gene expression (11.1 ng/mL). In contrast, the ratios of IC50 of Ara‐C divided by that of clofarabine for the cell lines with higher *DCK* gene expression (median ratio; 10.4) were significantly lower (*P *=* *0.0024) than those for the cell lines with lower *DCK* gene expression (30.2; Fig. 7D).

### Association of gene expression of regulatory factors with clofarabine sensitivity

**Figure 8:**
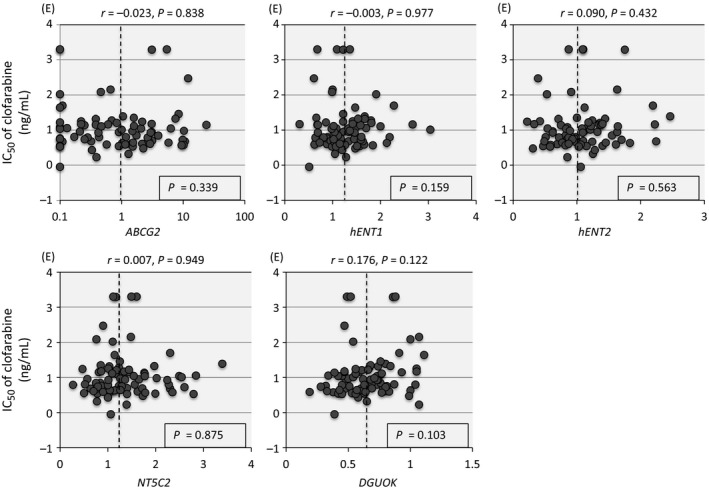
Association of clofarabine sensitivity with gene expression of *ABCG2* (*BCRP1*), *hENT1*,* hENT2*,* NT5C2,* and *DGUOK* in 79 BCP‐ALL cell lines. In each panel, vertical axis indicates log IC50 value of clofarabine, while horizontal axis indicates relative gene expression level. The correlation coefficient and *P* value are indicated at the top of each panel. Dotted line indicates median gene expression level. *P* value in a Mann–Whitney test of IC50 between 40 and 39 cell lines with relatively higher and lower gene expressions, respectively, is indicated in box.

We analyze association between the gene expression levels of regulatory factors and the sensitivity to clofarabine in 79 cell lines (Fig. 8). However, none of *ABCG2*,* hENT1*,* hENT2*,* DGUOK*, and *NT5C2* was associated with clofarabine sensitivity.

## Discussion

In the present study, to verify the involvement of DCK in the antileukemic activity of Ara‐C against BCP‐ALL, we knocked out DCK expression in an Ara‐C‐sensitive cell line using the CRISPR‐Cas9 system 14, 15. The knockout cells showed remarkable resistance to both Ara‐C and clofarabine but not to VCR and DNR, demonstrating that DCK activity is specifically indispensable for the antileukemic activities of Ara‐C and clofarabine in BCP‐ALL. Consistent with the critical role of DCK in the activation of Ara‐C, higher *DCK* gene expression was associated with higher Ara‐C sensitivity in 79 BCP‐ALL cell lines. In contrast, the association of *DCK* gene expression level with clofarabine sensitivity was marginal. The ratio of the IC50 of Ara‐C divided by that of clofarabine was significantly higher in cell lines with relatively lower rather than higher *DCK* gene expression, demonstrating that the antileukemic activity of clofarabine is relatively more potent in BCP‐ALL cells with relatively lower *DCK* gene expression. These observations suggest that clofarabine may be more effective to BCP‐ALL that shows relative resistance to Ara‐C due to low DCK expression (e.g., Ph+ALL and *MLL*+ALL).

A CpG island exists in the promoter region of the *DCK* gene 22, and several studies focused on the possible association of its methylation status with the sensitivity to nucleoside analogs in tumor samples. Leegwater et al. 27 analyzed a T‐ALL cell line and its cladribine‐ and clofarabine‐resistant sublines by sequencing genomic DNA treated with bisulfite; however, methylation was not observed in the resistant sublines as well as in the parental cell line. Ding et al. 28 analyzed 30 liver cancer cell lines by methylation‐specific PCR; however, methylation was not detected. Peters et al. 29 analyzed 10 cancer cell lines, including eight leukemia cell lines, by methylation‐specific PCR of four potential SP1 binding sites 30, and found that one SP1 site was weakly methylated in three cell lines; however, the remaining three sites were not methylated in all of the 10 cell lines. These previous reports suggested that an association of the methylation of the *DCK* gene promoter with sensitivity to nucleoside analogs in tumor cell lines is unlikely. However, direct sequencing and methylation‐specific PCR of the bisulfite‐treated genomic DNA in previous studies displayed limitations in terms of sensitivity and quantitative performance. Thus, in the present study, we applied a next‐generation DNA sequencing platform to precisely analyze the methylation status of 79 BCP‐ALL cell lines. We demonstrated that the % methylation was <5% at all 33 CG dinucleotide sites in the approximately 250‐bp region of the *DCK* gene promoter, which was almost identical to the region that was analyzed in the previous reports. Our observations clearly demonstrate that *DCK* gene silencing by methylation is not the mechanism for Ara‐C resistance, at least in BCP‐ALL cell lines.

Recently, next‐generation sequencing analyses of relapsed childhood ALL samples revealed somatic mutations in critical genes for drug metabolism, suggesting mechanisms for acquired drug‐resistant phenotypes; mutations in *NT5C2* 31, 32, 33 and *PRPS1* genes 34 have been reported to be involved in the specific resistance to 6‐mercaptopurine in relapsed ALL. As our analyses demonstrated that the *DCK* gene is critically involved in the antileukemic activity of Ara‐C, and more than half of the cell lines were established from relapsed ALL patients, we speculated that somatic mutations in the coding exons of the *DCK* gene may be associated with Ara‐C resistance in some BCP‐ALL cell lines. However, mutations were not observed in 79 BCP‐ALL cell lines, demonstrating that the acquisition of a somatic mutation in the coding exons of the *DCK* gene is uncommon as a mechanism for Ara‐C resistance.

Lamba et al. 23 reported three nonsynonymous SNPs of the *DCK* gene rs66878317 (I24V), rs66472932 (A119G), and rs67437265 (P122S). Our sequence analyses of the coding exons of the *DCK* gene identified a variant allele at rs67437265, but not at rs66878317 and rs66472932, in which variant allele frequencies were reported to be 0% in the normal Japanese population in the database of the 1000‐genome project. Lamba et al. 23 reported that mutant recombinant DCK protein with P122S showed approximately 40% DCK activity, with reduced *K*_*m*_ and *V*_*max*_ in a substrate kinetic study. Epstein–Barr virus‐transformed lymphoblast cell lines from individuals with a heterozygous mutant allele demonstrated significantly lower DCK activity compared with cell lines from homozygous wild‐type subjects. However, it is unclear whether reduced DCK activity due to a variant allele of rs67437265 is subsequently associated with reduced sensitivity to Ara‐C. In the present study, eight cell lines and one cell line showed a heterozygous and homozygous variant allele, respectively. The allele frequency in 72 BCP‐ALL cell lines established from Japanese patients was almost identical to that in the normal Japanese population. Of note, there was no significant difference in the IC50 values of Ara‐C between nine cell lines with a variant allele and 63 cell lines with a homozygous wild‐type allele. These observations suggest that the impact of a variant allele of rs67437265 on Ara‐C sensitivity may be less significant than the impact of *DCK* gene expression on Ara‐C sensitivity in BCP‐ALL cell lines.

Although Ara‐CMP is dephosphorylated by NT5C2 1, association of *NT5C2* gene expression with Ara‐C sensitivity was not clear in 79 PCB‐ALL cell lines. Gene expression of *ABCG2*, an efflux membrane transporter 24, *hENT2*, an uptake transporter 25, and *DGUOK,* an activating factor 26, was not associated with Ara‐C sensitivity. Unexpectedly, higher gene expression of *hENT1*, an uptake transporter 1, was associated with Ara‐C resistance. In our Ara‐C sensitivity assay, cells were cultured in the presence of Ara‐C for 72 h. One possible interpretation of this inverse association between *hENT1* gene expression and Ara‐C sensitivity is that significance of hENT1 in Ara‐C sensitivity might be substantially negligible in comparison with that of DCK, when hENT1 is expressed above a certain level. In this context, gene expression level of *hENT1* is somehow interrelated with that of *DCK* in BCP‐ALL cell lines. Indeed, gene expression level of *hENT1* was relatively lower in the cell lines with higher *DCK* gene expression than that in the cell lines with lower *DCK* gene expression (*P* = 0.078 in a Mann–Whitney test). Sensitivity to clofarabine was not associated with any of five factors tested.

Sensitivity to Ara‐C was significantly associated with the types of translocation. BCP‐ALL cell lines with poor prognostic translocations, such as Ph1, *MLL*r, and t(17;19), were significantly more resistant to Ara‐C than the cell lines with favorable translocations such as t(12;21) and t(1;19). The gene expression of *DCK* was significantly lower in t(17;19)‐ALL cell lines consistent with their Ara‐C resistance, but not in Ph+ALL and *MLL*+ALL cell lines despite their Ara‐C resistance. These observations suggested that low *DCK* gene expression may not be the main mechanism for Ara‐C resistance in Ph+ALL and *MLL*+ALL cell lines. In contrast to Ara‐C, clofarabine showed similar antileukemic activity against BCP‐ALL cell lines with poor prognostic translocations as well as those with favorable translocations, suggesting that clofarabine treatment may be more beneficial for patients with poor prognostic translocations than patients with favorable translocations.

## Authorship

M.H., K.M., and Y.T. performed the research, analyzed the data, and wrote the paper; T.I. designed the research study, performed the research, analyzed the data, and wrote the paper as principal investigator; K.K., M.A., A.W., S.S., T.S., H.O., K.A., and K.G. performed the research; H.G., M.M., and S.I. contributed essential cell lines; E.S. designed the research study; K.S. supervised the project; and all authors contributed to the final draft.

## Conflict of Interest

The authors declare no competing financial interests.

## Supporting information


**Figure S1**. Electrophoresis of genomic PCR products in Ara‐C–resistant clones of the CRISPR–Cas9 treansfected KOPN41.
**Figure S2**. Chromatograms of wild‐type and DCK knockout clone of KOPN41 cultured in the absence or presence of 20 μM Ara‐C.
**Figure S3**. Ara‐C specific resistance in DCK knockout clones.
**Figure S4**. Sequencing of RT–PCR products in codon 122 of the *DCK* gene.

## References

[1] Lamba, J. K. 2009 Genetic factors influencing cytarabine therapy. Pharmacogenomics 10:1657–1674. 19842938 10.2217/pgs.09.118 PMC2828057

[2] Dumontet, C. , K. Fabianowska‐Majewska , D. Mantincic , E. Callet Bauchu , I. Tigaud , V. Gandhi , et al. 1999 Common resistance mechanisms to deoxynucleoside analogues in variants of the human erythroleukaemic line K562. Br. J. Haematol. 106:78–85. 10444166 10.1046/j.1365-2141.1999.01509.x

[3] Stegmann, A. P. , W. H. Honders , R. Willemze , V. W. Ruiz van Haperen , and J. E. Landegent . 1995 Transfection of wild‐type deoxycytidine kinase (dck) cDNA into an AraC‐ and DAC‐resistant rat leukemic cell line of clonal origin fully restores drug sensitivity. Blood 85:1188–1194. 7532033

[4] Galmarini, C. M. , X. Thomas , K. Graham , A. El Jafaari , E. Cros , L. Jordheim , et al. 2003 Deoxycytidine kinase and cN‐II nucleotidase expression in blast cells predict survival in acute myeloid leukaemia patients treated with cytarabine. Br. J. Haematol. 122:53–60. 12823345 10.1046/j.1365-2141.2003.04386.x

[5] Stammler, G. , F. Zintl , A. Sauerbrey , and M. Volm . 1997 Deoxycytidine kinase mRNA expression in childhood acute lymphoblastic leukemia. Anticancer Drugs 8:517–521. 9215616 10.1097/00001813-199706000-00015

[6] Nowak, D. , N. L. Liem , M. Mossner , M. Klaumünzer , R. A. Papa , V. Nowak , et al. 2015 Variegated clonality and rapid emergence of new molecular lesions in xenografts of acute lymphoblastic leukemia are associated with drug resistance. Exp. Hematol. 43:32–43. e31–35. 25450514 10.1016/j.exphem.2014.09.007 PMC5894481

[7] Stam, R. W. , M. L. den Boer , J. P. Meijerink , M. E. Ebus , G. J. Peters , P. Noordhuis , et al. 2003 Differential mRNA expression of Ara‐C‐metabolizing enzymes explains Ara‐C sensitivity in MLL gene‐rearranged infant acute lymphoblastic leukemia. Blood 101:1270–1276. 12406912 10.1182/blood-2002-05-1600

[8] Hijiya, N. , E. Barry , and R. J. Arceci . 2012 Clofarabine in pediatric acute leukemia: current findings and issues. Pediatr. Blood Cancer 59:417–422. 22354543 10.1002/pbc.24112

[9] Zhenchuk, A. , K. Lotfi , G. Juliusson , and F. Albertioni . 2009 Mechanisms of anti‐cancer action and pharmacology of clofarabine. Biochem. Pharmacol. 78:1351–1359. 19576186 10.1016/j.bcp.2009.06.094

[10] Jeha, S. , V. Gandhi , K. W. Chan , L. McDonald , I. Ramirez , R. Madden , et al. 2004 Clofarabine, a novel nucleoside analog, is active in pediatric patients with advanced leukemia. Blood 103:784–789. 14551141 10.1182/blood-2003-06-2122

[11] Jeha, S. , P. S. Gaynon , B. I. Razzouk , J. Franklin , R. Kadota , V. Shen , et al. 2006 Phase II study of clofarabine in pediatric patients with refractory or relapsed acute lymphoblastic leukemia. J. Clin. Oncol. 24:1917–1923. 16622268 10.1200/JCO.2005.03.8554

[12] Hijiya, N. , B. Thomson , M. S. Isakoff , L. B. Silverman , P. G. Steinherz , M. J. Borowitz , et al. 2011 Phase 2 trial of clofarabine in combination with etoposide and cyclophosphamide in pediatric patients with refractory or relapsed acute lymphoblastic leukemia. Blood 118:6043–6049. 21967976 10.1182/blood-2011-08-374710 PMC3731655

[13] Escherich, G. , U. Zur Stadt , M. Zimmermann , M. A. Horstmann , and CoALL study group . 2013 Clofarabine in combination with pegylated asparaginase in the frontline treatment of childhood acute lymphoblastic leukaemia: a feasibility report from the CoALL 08‐09 trial. Br. J. Haematol. 163:240–247. 23937310 10.1111/bjh.12520

[14] Cong, L. , F. A. Ran , D. Cox , S. Lin , R. Barretto , N. Habib , et al. 2013 Multiplex genome engineering using CRISPR/Cas systems. Science 339:819–823. 23287718 10.1126/science.1231143 PMC3795411

[15] Mali, P. , L. Yang , K. M. Esvelt , J. Aach , M. Guell , J. E. DiCarlo , et al. 2013 RNA‐guided human genome engineering via Cas9. Science 339:823–826. 23287722 10.1126/science.1232033 PMC3712628

[16] Goto, H. , T. Naruto , R. Tanoshima , H. Kato , T. Yokosuka , M. Yanagimachi , et al. 2009 Chemo‐sensitivity in a panel of B‐cell precursor acute lymphoblastic leukemia cell lines, YCUB series, derived from children. Leuk. Res. 33:1386–1391. 19157546 10.1016/j.leukres.2008.12.003

[17] Kang, J. , R. R. Kisenge , H. Toyoda , S. Tanaka , J. Bu , E. Azuma , et al. 2003 Chemical sensitization and regulation of TRAIL‐induced apoptosis in a panel of B‐lymphocytic leukaemia cell lines. Br. J. Haematol. 123:921–932. 14632785 10.1046/j.1365-2141.2003.04699.x

[18] Minegishi, M. , S. Tsuchiya , N. Minegishi , and T. Konno . 1987 Establishment of five human malignant non‐T lymphoid cell lines and mixed lymphocyte‐tumor reaction. Tohoku J. Exp. Med. 151:283–292. 2954268 10.1620/tjem.151.283

[19] Zhang, X. , T. Inukai , K. Hirose , K. Akahane , I. Kuroda , H. Honna‐Oshiro , et al. 2012 Oncogenic fusion E2A‐HLF sensitizes t(17;19)‐positive acute lymphoblastic leukemia to TRAIL‐mediated apoptosis by upregulating the expression of death receptors. Leukemia 26:2483–2493. 22743623 10.1038/leu.2012.139

[20] Bachmann, P. S. , R. G. Piazza , M. E. Janes , N. C. Wong , C. Davies , A. Mogavero , et al. 2010 Epigenetic silencing of BIM in glucocorticoid poor‐responsive pediatric acute lymphoblastic leukemia, and its reversal by histone deacetylase inhibition. Blood 116:3013–3022. 20647567 10.1182/blood-2010-05-284968

[21] Huang, M. , K. Miyake , K. Kagami , M. Abe , T. Shinohara , A. Watanabe , et al. 2017 Lack of association between deletion polymorphism of BIM gene and in vitro drug sensitivity in B‐cell precursor acute lymphoblastic leukemia. Leuk. Res. 60:24–30. 28641145 10.1016/j.leukres.2017.06.003

[22] Song, J. J. , S. Walker , E. Chen , E. E. Johnson 2nd , J. Spychala , T. Gribbin , et al. 1993 Genomic structure and chromosomal localization of the human deoxycytidine kinase gene. Proc. Natl Acad. Sci. USA 90:431–434. 8421671 10.1073/pnas.90.2.431 PMC45676

[23] Lamba, J. K. , K. Crews , S. Pounds , E. G. Schuetz , J. Gresham , V. Gandhi , et al. 2007 Pharmacogenetics of deoxycytidine kinase: identification and characterization of novel genetic variants. J. Pharmacol. Exp. Ther. 323:935–945. 17855478 10.1124/jpet.107.128595

[24] Fukuda, Y. , and J. D. Schuetz . 2012 ABC transporters and their role in nucleoside and nucleotide drug resistance. Biochem. Pharmacol. 83:1073–1083. 22285911 10.1016/j.bcp.2011.12.042 PMC3319017

[25] Endo, Y. , T. Obata , D. Murata , M. Ito , K. Sakamoto , M. Fukushima , et al. 2007 Cellular localization and functional characterization of the equilibrative nucleoside transporters of antitumor nucleosides. Cancer Sci. 98:1633–1637. 17711502 10.1111/j.1349-7006.2007.00581.x PMC11159219

[26] Zhu, C. , M. Johansson , J. Permert , and A. Karlsson . 1998 Enhanced cytotoxicity of nucleoside analogs by overexpression of mitochondrial deoxyguanosine kinase in cancer cell lines. J. Biol. Chem. 273:14707–14711. 9614068 10.1074/jbc.273.24.14707

[27] Leegwater, P. A. , R. A. De Abreu , and F. Albertioni . 1998 Analysis of DNA methylation of the 5′ region of the deoxycytidine kinase gene in CCRF‐CEM‐sensitive and cladribine (CdA)‐ and 2‐chloro‐2′‐arabino‐fluoro‐2′‐deoxyadenosine (CAFdA)‐resistant cells. Cancer Lett. 130:169–173. 9751270 10.1016/s0304-3835(98)00131-1

[28] Ding, S. , B. D. Gong , J. Yu , J. Gu , H. Y. Zhang , Z. B. Shang , et al. 2004 Methylation profile of the promoter CpG islands of 14 “drug‐resistance” genes in hepatocellular carcinoma. World J. Gastroenterol. 10:3433–3440. 15526362 10.3748/wjg.v10.i23.3433 PMC4576224

[29] Peters, G. J. , J. Hodzic , B. Ortega , E. Giovannetti , A. D. Adema , R. Broekhuizen , et al. 2010 Methylation specific PCR to characterize methylation of the promoter of deoxycytidine kinase. Nucleosides Nucleotides Nucleic Acids 29:408–413. 20544528 10.1080/15257771003730078

[30] Chen, E. H. , E. E. Johnson 2nd , S. M. Vetter , and B. S. Mitchell . 1995 Characterization of the deoxycytidine kinase promoter in human lymphoblast cell lines. J. Clin. Invest. 95:1660–1668. 7706474 10.1172/JCI117841 PMC295671

[31] Ma, X. , M. Edmonson , D. Yergeau , D. M. Muzny , O. A. Hampton , M. Rusch , et al. 2015 Rise and fall of subclones from diagnosis to relapse in pediatric B‐acute lymphoblastic leukaemia. Nat. Commun. 6:6604. 25790293 10.1038/ncomms7604 PMC4377644

[32] Meyer, J. A. , J. Wang , L. E. Hogan , J. J. Yang , S. Dandekar , J. P. Patel , et al. 2013 Relapse‐specific mutations in NT5C2 in childhood acute lymphoblastic leukemia. Nat. Genet. 45:290–294. 23377183 10.1038/ng.2558 PMC3681285

[33] Tzoneva, G. , A. Perez‐Garcia , Z. Carpenter , H. Khiabanian , V. Tosello , M. Allegretta , et al. 2013 Activating mutations in the NT5C2 nucleotidase gene drive chemotherapy resistance in relapsed ALL. Nat. Med. 19:368–371. 23377281 10.1038/nm.3078 PMC3594483

[34] Li, B. , H. Li , Y. Bai , R. Kirschner‐Schwabe , J. J. Yang , Y. Chen , et al. 2015 Negative feedback‐defective PRPS1 mutants drive thiopurine resistance in relapsed childhood ALL. Nat. Med. 21:563–571. 25962120 10.1038/nm.3840 PMC4670083

