# Supplementary material for: Clofarabine exerts antileukemic activity against cytarabine‐resistant B‐cell precursor acute lymphoblastic leukemia with low deoxycytidine kinase expression
Source: Cancer Med. 2018 Feb 23;7(4):1297–316. doi: 10.1002/cam4.1323 (PMC5911575; doi:10.1002/cam4.1323)
Supplement: Supplementary file 1 — Figure S1. Electrophoresis of genomic PCR products in Ara‐C–resistant clones of the CRISPR–Cas9 treansfected KOPN41. Figure S2. Chromatograms of wild‐type and DCK knockout clone of KOPN41 cultured in the absence or presence of 20 μM Ara‐C. Figure S3. Ara‐C specific resistance in DCK knockout clones. Figure S4. Sequencing of RT–PCR products in codon 122 of the DCK gene. [file CAM4-7-1297-s001.docx]

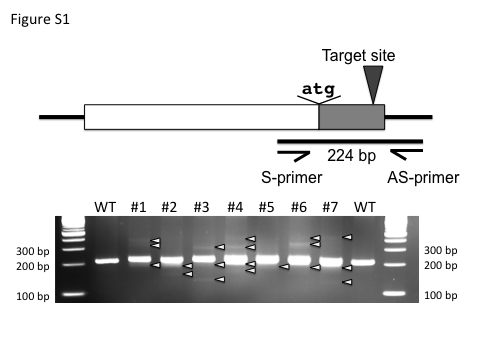


**Figure S1** Electrophoresis of genomic PCR products in Ara-C–resistant clones of the CRISPR–Cas9 treansfected KOPN41. The upper panel indicates schematic representation of target site with primers for PCR. The lower panel indicates the electrophoresis of the PCR products of seven Ara-C-resistant clones (#1 to #7) and wild-type cells (WT) of KOPN41. Arrowheads indicate extra products with abnormal size.

**Figure S2** Chromatograms of wild-type and DCK knockout clone of KOPN41 cultured in the absence or presence of 20 μM Ara-C. Green arrowhead indicates the peek of Ara-CTP, while red arrowhead in the box indicates the peek of ^13^C-UTP added as an internal standard.


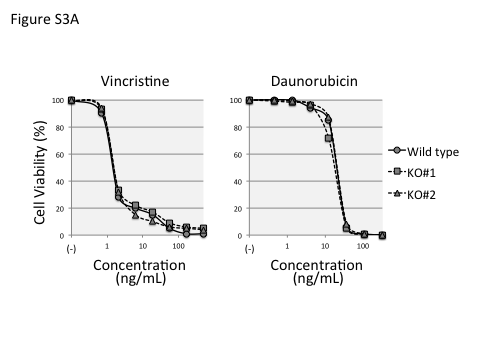

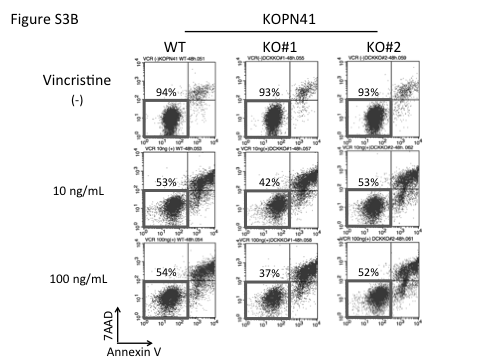

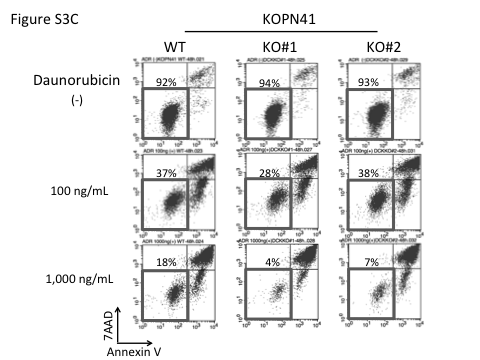


**Figure S3** Ara-C specific resistance in DCK knockout clones. (A) Dose-response curves for vincristine (left) and daunorubicin (right) in wild-ype and DCK knockout (KO) clones of KOPN41. Representative results of alamarBlue assays are indicated. The vertical axis indicates the % viability and the horizontal axis indicates the log concentrations of vincristine (left) and daunorubicin (right). (B and C) The induction of apoptotic cell death by vincristine (B) and daunorubicin (C). Cells were cultured in the absence or presence of 30 and 100ng/mL of vincristine (B) and 100 and 1,000ng/mL of daunorubicin (C) for 48 hours, and analysed with Annexin V binding (horizontal axis) and a 7AAD stain (vertical axis) using flow cytometry. The percentages of living cells are indicated.


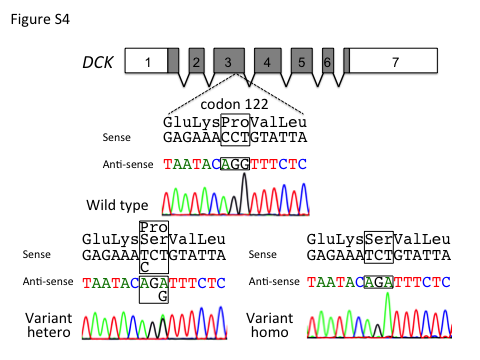


**Figure S4** Sequencing of RT–PCR products in codon 122 of the *DCK* gene. Representative sequences of a wild-type, a heterozygous variant genotype, and a homozygous variant genotype of the rs67437265 are indicated.
